# Social defeat stress induces an anxiety-like outcome in male prairie voles (*Microtus ochrogaster*)

**DOI:** 10.1093/oons/kvae012

**Published:** 2024-11-23

**Authors:** Minerva Rodriguez, Anapaula Themann, Daniel E Calvo, Jessica A Garcia, Omar Lira, Israel Garcia-Carachure, Sergio D Iñiguez

**Affiliations:** Department of Psychology, The University of Texas at El Paso, El Paso, TX 79968; Department of Psychology, The University of Texas at El Paso, El Paso, TX 79968; Department of Psychology, The University of Texas at El Paso, El Paso, TX 79968; Department of Psychology, The University of Texas at El Paso, El Paso, TX 79968; Department of Psychology, The University of Texas at El Paso, El Paso, TX 79968; Department of Psychology, The University of Texas at El Paso, El Paso, TX 79968; Department of Psychology, The University of Texas at El Paso, El Paso, TX 79968

**Keywords:** anxiety, elevated plus maze, light/dark box, prairie vole, stress model, social defeat

## Abstract

Anxiety-related illnesses constitute one of the leading causes of disability across the globe. Consequently, the need for validated preclinical models to uncover the etiology of anxiety phenotypes remains essential. Given the link between social stress experience and the manifestation of anxiogenic-like outcomes, we evaluated whether social defeat stress (SDS) reduces open-space exploratory behavior in prairie voles (*Microtus ochrogaster*). Thus, we exposed adult sexually-naïve male voles to 10 consecutive days of SDS episodes and evaluated responses to the anxiogenic environment of the light/dark box test or the elevated plus-maze, 24 hours later. We found that, when compared to non-stressed controls, SDS-exposed voles displayed longer latency to enter the light compartment of the light/dark box. Similarly, on the elevated plus-maze, SDS-exposed voles displayed decreases in the number of entries into the open arms, while spending more time in the closed arms of the maze. No differences in locomotor activity were noted between the experimental groups. Collectively, these data indicate that chronic SDS exposure induces anxiety-like responses in adult male prairie voles, thus, providing a preclinical model for the study of social stress-induced anxiogenic phenotypes.

## INTRODUCTION

Despite the high prevalence of anxiety-related disorders worldwide [1] their etiology and treatment are not well understood [2]. Exposure to chronic social stress, whether physical and/or psychological, is a well-recognized risk factor for the development of anxiety-related syndromes [3, 4]. For this reason, identifying the characteristics that precipitate vulnerability to anxiety, using social stress-related animal models [5, 6] is critical for uncovering the underlying biological mechanisms that mediate anxiogenic pathologies, which in turn can provide a platform for new therapeutic approaches that effectively manage and treat the disease [7].

The social defeat stress (SDS) preclinical paradigm [8] is a validated approach for the study of behaviors associated with stress-induced illnesses [9, 10]. Indeed, SDS-exposed rodents exhibit decreases in open-space exploratory behavior across numerous experimental endpoints validated for the study of anxiety [11–13] – outcomes that are reversed by the administration of medications with anxiolytic properties in humans [6, 14]. More importantly, SDS produces adaptations within the brain of rodents [15] that are analogous to those observed in postmortem human tissue of individuals who suffered from mood-related illnesses [16–19]. Because of the dire need to identify the biological mechanisms underlying anxiety-related disorders, existing animal models need to be refined to capture the etiology of social stress-induced anxiety [13, 20].

Within the field of neuroscience, the prairie vole (*Microtus ochrogaster*) has surfaced as a model to investigate the behavioral and biological consequences of stress-induced sequalae [21]. For example, chronic (10 days) exposure to SDS in prairie voles [22], like in mice [12, 23, 24], results in a behavioral profile that resembles a depression-related outcome [5]. Specifically, SDS-exposed voles display (1) reductions in body weight, (2) spatial memory impairment, along with (3) decreases of social behavior and (4) sucrose preference (i.e. an anhedonia-like outcome). However, whether chronic SDS exposure (i.e. 10 episodes) induces sequalae relevant to anxiety-related behavior, in male prairie voles specifically, has not been evaluated. For this reason, here, we exposed adult male prairie voles to 10 consecutive days of SDS and assessed open-space exploratory behaviors adopting standard validated measures to study anxiety-like responses at the preclinical level [25, 26]. We hypothesized that exposure to SDS would reduce open-space exploratory behavior (as described below), thus, uncovering a chronic stress-induced anxiogenic effect [27].

## METHODS

### Animals

Laboratory-reared adult (80–120 days of age) male prairie voles (*M. ochrogaster*) were used in the present investigation. The voles originated from a wild stock trapped near Urbana, IL (USA). After weaning at 21-days of age, they were housed in same-sex sibling pairs in a colony room maintained on a 14:10 hour light/dark cycle (light on at 0700 hours) under temperature-controlled conditions (20–21°C). Voles were housed in standard polysulfonate rat cages (Ancare, Bellmore, NY) with pine chip bedding, with unrestricted access to water and food (rabbit chow). The experimental approach was conducted in compliance with ARRIVE guidelines [28] and approval of the *Institutional Animal Care and Use Committee* at The University of Texas at El Paso (UTEP).

### Aggression screening in sexually experienced male voles

As previously described, sexually experienced male voles underwent three consecutive days of an aggression-selection process [22]. Briefly, the territorialized home-cage (10½ in X 19 in X 6⅛ in; N40 cage; Ancare, Bellmore, NY) of an active breeding pair-bonded couple (male and female) was divided in two separate equal-size compartments by inserting a fitted perforated Plexiglass-divider into the center of the cage. Before starting the aggressive behavior screening process, the paired-couple (with potential offspring) would be physically separated (one on each side of the cage) and were allowed to acclimate for 1 minute. Afterwards, a sexually naïve male screener-vole (not part of the experiment) was placed in the compartment of the paired-male vole, and the time (seconds) to physically attack the screener conspecific was recorded during a 3 minute session. At the end of the aggression-screening session, the screener-vole was removed and checked for potential injuries and returned to its home cage. No behaviors were recorded from the screener vole. Immediately after, the Plexiglass-divider was removed from the cage re-uniting the pair-bonded male-and-female couple (and potential pups). Sexually experienced male paired-voles with similar and consistent levels of aggression (physically attacking within 30 seconds) across the 3-consecutive days were selected as aggressors for the SDS experiment. Around 70% of the male sexually-experienced voles displayed consistent aggression and thus were selected as aggressors to physically defeat the experimental animals [22, 29], as described below.

### Social defeat stress (SDS)

**Figure 1:**
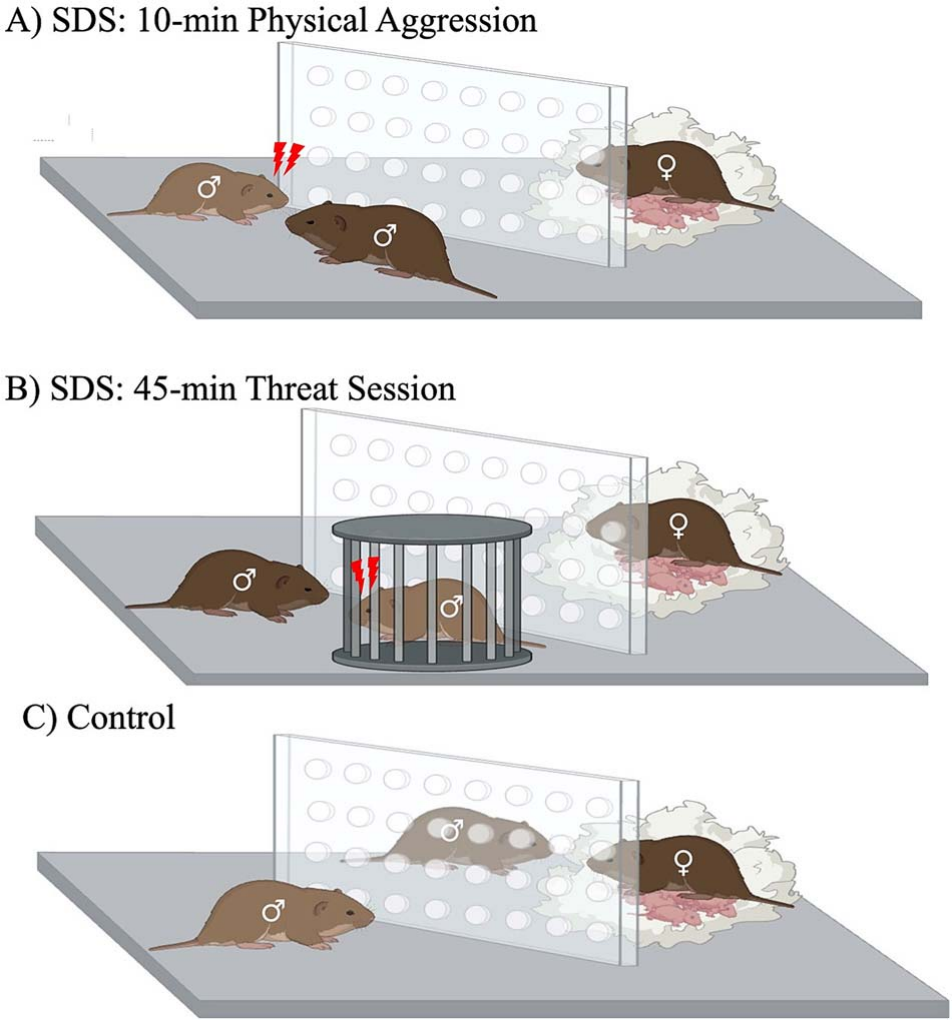
Schematic of the social defeat stress (SDS) protocol. (**A**) the experimental vole is forced to intrude the territorialized compartment of a pair-bonded aggressor’s home-cage, resulting in a physically aggressive encounter (10 minutes). (**B**) after the physical defeat encounter, the SDS-exposed vole is placed inside a holding cage for an additional 45-minute threat-session in the presence of the aggressor. (**C**) Non-stressed control (CON) voles were housed in analogous cage/housing conditions (with a pair-bonded male/female couple and their potential offspring) but never experienced social conflict.

SDS was conducted as previously described [22]. Plexiglass perforated dividers were added to the home cage of pair-bonded (actively breeding) aggressors one day prior to the start of the experiment; partitioning the home-cage into two equal size compartments (additional equipment details in [30]). For each defeat episode, the pair-bonded male aggressor was moved to the opposite side of the compartment from their respective pair-bonded female. The pair-bonded female (and potential pups) remained in the same compartment throughout the SDS episode (opposite side), yet never physically interacted with the experimental intruder. Adult sexually-naïve experimental male voles (i.e. intruders) were randomly assigned to experience SDS episodes or were housed in non-stressed control (CON) conditions. Experimental voles assigned to the SDS condition were placed into the compartment of the aggressor for up to 10 minutes; resulting in a combative physical encounter (Fig. 1A). To prevent injury, in the event of overly-antagonistic combat, the physical encounter between the voles would be interrupted for a few seconds by distracting the aggressor-vole with a standard plastic ruler (24 cm in length). After the 10-minute physical bout, the experimental intruder-vole was placed into a holding container for an additional 45-minute threat-session in the presence of the aggressor (Fig. 1B). After the 45-minute non-physical threat-session, the male aggressor was returned to the compartment with its female partner (opposite side of cage). At this point, the experimental SDS intruder-vole was released from the holding container, examined for potential injuries, and remained there for 24 hours (until the next SDS episode with a different aggressor). This procedure was adopted to ensure that SDS-exposed voles were defeated/stressed by a different aggressor-vole every day (across each of the 10 consecutive days). CON sexually-naïve male prairie voles were housed in similar conditions (i.e. in the compartment adjacent to a novel bonded male-and-female couple for 10 consecutive days, using the same holding container to transfer them across cages each day) but never experienced a physical confrontation or non-physical threat-session with the male pair-bonded aggressor (Fig. 1C). After the 10th SDS episode, separate groups of experimental animals (CON and SDS) were housed individually in standard laboratory cages (7½ in X 11½ in X 5 in; N10 cage; Ancare, Bellmore, NY). Twenty-four hours later, CON and SDS voles were evaluated for anxiety-related responses, as described below. Behavioral outcomes were recorded/evaluated via an automated computer video-tracking system (EthovisionXT, Noldus, Leesburg, VA). All behavioral testing was conducted during the light cycle period of the day (between 1000–1400 hours).

### Light/dark box (LDB)

The LDB test is commonly adopted to evaluate anxiety-related responses [25, 31]; theoretically, by capturing the apprehension created between a rodent’s innate motivation to explore novel environments and avoiding open spaces [25, 32–34]. The LDB apparatus consisted of two equal size interconnected chambers (20 cm in width × 40 cm in length × 35 cm in height) made of Plexiglass. One chamber was black (with a covered enclosure-lid), while the other one was white and uncovered (Model 63 101; Stoelting, Wood Dale, IL). Behavioral testing was conducted in a quiet (< 40 dB) behavioral testing room under red-light conditions (~ 30 lx). Experimental animals were initially placed in the dark/covered compartment and allowed to move freely between the two chambers for a total of 5 minutes. Latency (seconds) to enter the open-space light side, as well as the total time (seconds) spent in the light compartment during the 5 minutes session were the dependent variables. Longer latency to initially enter the light side (i.e. white compartment) was inferred as an anxiogenic-related response.

### Elevated plus maze (EPM)

The EPM apparatus was purchased from Stoelting Co. (Model 60140; Wood Dale, IL) and consisted of two perpendicular runways in the shape of a plus (+) sign (gray in color and elevated 40 cm from the floor). Each runway was 5 cm wide × 35 cm long, with a central 5 × 5 cm central connecting area. The closed arms runway had walls on each side (15 cm in height) while the open arms runway did not (making the height of the maze visible to the animal). At the start of the experiment, voles were placed in the center area, and were allow to explore the maze for a total of 5 minutes, as previously described [14]. Behavior was conducted in a quiet (< 40 dB) behavioral testing room under red-light conditions (~30 lx). The total time spent in the arms of the maze (seconds), and total number of entries into the open arms, were the dependent variables. The center of the maze (5 × 5 cm connecting area) was not categorized to be part of either the open or closed arm zones, and thus was not analyzed. Decreases in EPM exploration were interpreted as an anxiogenic-like effect as traditionally described across the literature [13, 14, 35].

**Figure 2:**
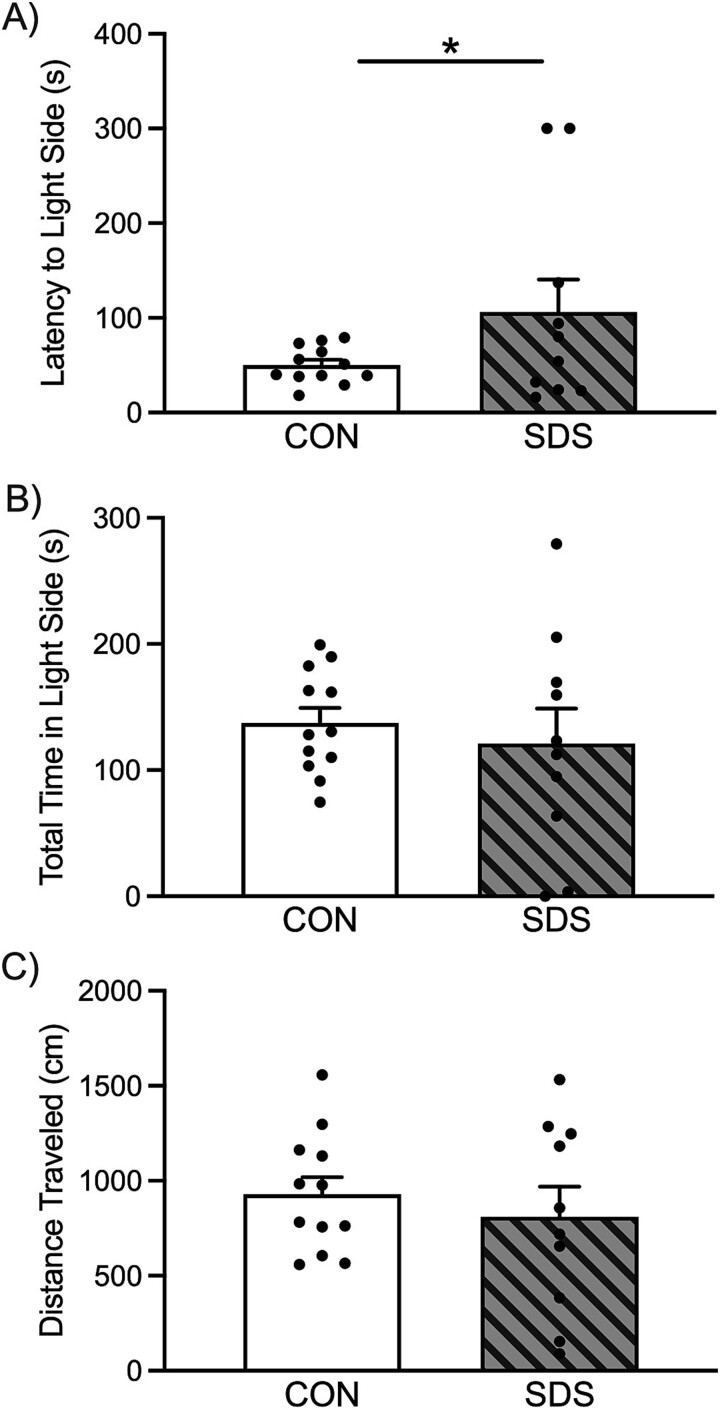
Effects of social defeat stress (SDS) on light/dark box (LDB) performance in adult sexually-naïve male prairie voles. (**A**) When compared to the non-stressed control (CON) group (n = 12), the SDS-exposed voles (n = 10) displayed longer latency (seconds) to enter the light compartment of the light/dark box. (**B**) No differences in the total time (seconds) spent in the light compartment were noted between the groups. Likewise, (**C**) no alterations in locomotor activity (i.e. distance traveled in cm) were detected between experimental conditions. Data are presented as mean + SEM. ^*^P ≤ 0.05. s, seconds.

### Statistical analysis

Voles were randomly assigned to the different stress/housing conditions (CON or SDS). To avoid carryover effects, separate groups of animals were used for the two behavioral tasks implemented (i.e. LDB and EPM). As a result of the death of two animals in the SDS condition, per experimental cohort, there were unequal number of animals between the SDS and CON conditions. Data were analyzed adopting Student’s t-test. Results are presented as mean + standard error of the mean (+SEM) and statistical significance was defined as *P* ≤ 0.05 (see Table 1 for statistical values, number of animals per experimental condition, and effect sizes). No outliers were detected in our sets of data according to Grubbs’ tests. Figures and statistical analyses were performed using GraphPad Prism (Boston, MA; version 9).

**Table 1:** Experimental groups and statistical values

**Figure**	**Behavior**	**Statistical Test**	**Dependent Variable**	**n**	**Statistical Value**	***P*-value**	**η**_**p**_^**2**^
2A	Light/Dark Box (Cohort 1)	Student’s t-test	Latency to Light Side (s)	CON n = 12	t_(20)_ = 1.74	*P* = 0.04^*^	0.13
				SDS n = 10			
2B			Total Time in Light Side (s)	CON n = 12	t_(20)_ = 0.57	*P* = 0.28	0.01
				SDS n = 10			
2C			Distance Traveled (cm)	CON n = 12	t_(20)_ = 0.67	*P* = 0.25	0.02
				SDS n = 10			
3A	Elevated Plus-Maze (Cohort 2)	Student’s t-test	Time in Closed Arms (s)	CON n = 7	t_(10)_ = 1.81	*P* = 0.05^*^	0.24
				SDS n = 5			
3B			Open Arm Entries	CON n = 7	t_(10)_ = 3.78	*P* = 0.01^*^	0.58
				SDS n = 5			
3C			Time in Open Arms (s)	CON n = 7	t_(10)_ = 0.88	*P* = 0.19	0.07
				SDS n = 5			
3D			Distance Traveled (cm)	CON n = 7	t_(10)_ = 0.12	*P* = 0.45	0.002
				SDS n = 5			
4	Body Weight (Cohort 1)	Student’s t-test	Total Weight (g)	CON n = 12	t_(20)_ = 1.70	*P* = 0.05^*^	0.12
				SDS n = 10			

**CON**, control; **SDS**, social defeat stress; **s**, seconds

^*^, *P*-value is less or equal to 0.05

**Figure 3:**
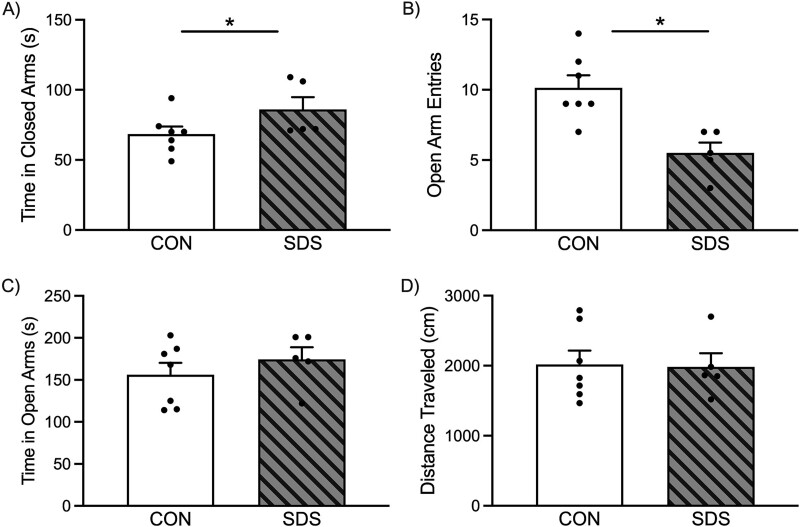
Effects of social defeat stress (SDS) on elevated plus maze (EPM) performance in adult sexually-naïve male prairie voles. (**A**) When compared to the non-stressed control (CON) group (n = 7), the SDS-exposed voles (n = 5) spent more time (seconds) in the closed arms of the EPM. Likewise, (**B**) SDS-exposed voles displayed lower number of entries into the open arms of the maze. (**C**) No differences in the total time (seconds) spent in the open arms were noted between the groups. (D) No differences in distance traveled between SDS and CON voles were noted during the 5-minute test. Data are presented as mean + SEM. ^*^P ≤ 0.05. s, seconds.

## RESULTS

### Exposure to chronic SDS induces anxiety-related behavior in the LDB test

Figure 2A displays the behavioral effects of SDS on the LDB test in adult sexually-naïve male prairie voles. When compared to CON’s (n = 12), voles exposed to SDS (n = 10) displayed increased latency (seconds) to enter the light compartment of the LDB (t_20_ = 1.74, *P* ≤ 0.05). Figure 2B shows that no differences in total time (seconds) spent in the light compartment were noted between the experimental groups (t_20_ = 0.57, *P* = 0.28). When assessing locomotor activity within the light compartment, Fig. 2C shows that no differences in distance traveled (cm) were observed between the CON and SDS groups (t_20_ = 0.67, *P* = 0.25).

### Chronic SDS induces an anxiogenic-like behavioral response in the EPM

Figure 3 shows the effects of SDS on exploratory behavior in the EPM. Specifically, when compared to non-stressed CON’s (n = 7), voles exposed to SDS (n = 5) spent more time within the closed arms of the maze (t_10_ = 1.81, *P* ≤ 0.05; Fig. 3A). This SDS-induced reduction in exploratory behavior was accompanied by lower open-arm entries (t_10_ = 3.78, *P* ≤ 0.05; Fig. 3B), without difference in total time spent in the open arms of the maze (Fig. 3C). No differences in total distance traveled (Fig. 3D) were noted between the groups (*P* = 0.12) during the 5-minute test.

### SDS decreases body weight

**Figure 4:**
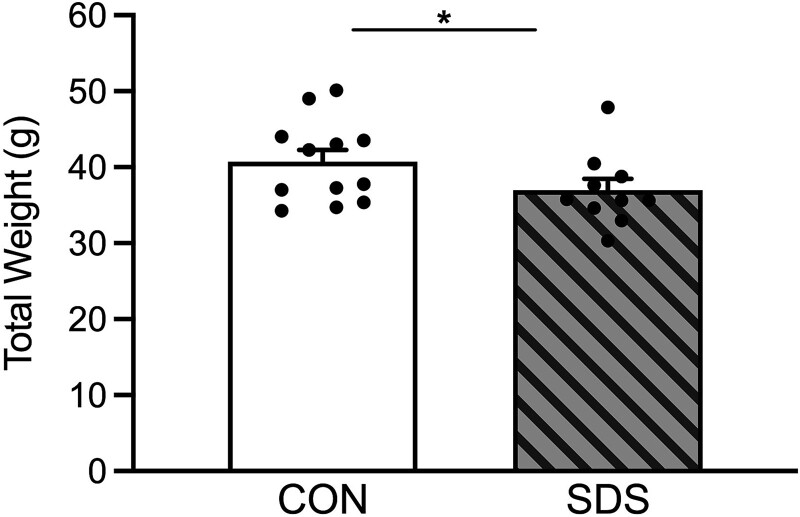
Effects of social defeat stress (SDS) on body weight (g) in adult sexually-naïve male prairie voles, 24 hours after the last SDS episode. When compared to the non-stressed control (CON) group (n = 12), the SDS-exposed voles (n = 10) displayed lower body weight (g). Data are presented as mean + SEM. ^*^P ≤ 0.05. g, grams.

Figure 4 displays the effects of chronic SDS on total body weight (g) in adult male prairie voles. As previously reported [22], 24 hours after the last stress episode (i.e. before behavioral testing) voles exposed to SDS displayed lower body weight, when compared to non-stressed CONs (t_20_ = 1.70, *P* ≤ 0.05).

## DISCUSSION

Anxiety-related pathologies constitute a significant disease burden throughout the world, particularly because they are highly comorbid with numerous psychiatric conditions, including major depression [36]. For this reason, it is essential to develop and/or refine preclinical models to uncover the etiology of anxiety-related phenotypes [14, 37]. Given the well-established tie between social stress history and the development of anxiogenic-like outcomes [38] we evaluated whether chronic SDS exposure reduces open-space exploratory behavior in adult male prairie voles. This approach was taken because reductions in exploratory behavior, post chronic stress exposure, is a validated anxiogenic-related phenotype across rodent species [25, 27, 33, 39].

Here, we show for the first time that 10 episodes of SDS (Fig. 1) results in decreases of open-space exploration in two separate tasks validated for the study of anxiety-related behavior. Specifically, SDS-exposed voles showed longer time (seconds) to initially enter and explore the light side of the LDB (Fig. 2A), while also entering with less frequency the open arms (while spending more time within the enclosed arms) of the EPM (Fig. 3A and B). Importantly, this anxiety-like response was not due to potential stress-induced changes in locomotor activity despite SDS-induced decreases in body weight (Fig. 4); which are commonly reported post SDS exposure in different rodent species [6, 15] including prairie voles [22]. This is likely the case since no differences between the groups were noted when evaluating distance traveled (cm) in the light side of the LDB (Fig. 2C) or total time spent in the EPM (Fig. 3D). Importantly, this SDS-induced behavioral response complements/expands prior literature in male prairie voles specifically; wherein 4-weeks of social isolation stress decreased total time spent in the light side of the LDB [26], highlighting that this task is a reliable measure to capture anxiogenic phenotypes in voles. Similarly, adult mandarin voles (of both sexes) exhibit decreases in exploration when evaluated in the LDB, open field, and EPM after 14 exposures to SDS [40, 41]. Importantly, we further show that 10 episodes of SDS induces anxiety-related behavior in the EPM, in a similar manner to 3 acute episodes, as reported in both male and female prairie voles [29]. Yet, chronic, but not acute, exposure to social stress is an important distinction to consider when validating animal models for the study of affect-related disorders, given that neither humans nor animals develop mood-related syndromes after an acute stressor [42]. Indeed, acute stress results in adaptive behavioral processes that lead to the survival of the organism [43], unlike the expression of maladaptive responses post chronic stress exposure [44]. Therefore, adopting chronic regimens of ethological stress, when validating preclinical models, is more likely to uncover the links underlying the development of anxiety and/or depressive-related syndromes [45]. Collectively, this suggests that chronic, but not acute, SDS in prairie voles provides robust face validity to this preclinical paradigm specifically; particularly for the study of comorbid anxiety (present results) and depression-related outcomes [22].

The neurobiological mechanisms that underlie the observed SDS-induced decrease in exploratory behavior in male prairie voles are not well understood. Although the literature on stress-induced anxiety-related behavior in prairie voles is limited [46, 47], various forms of stress increase plasma corticosterone in adult male prairie voles [48] while decreasing both EPM exploration and adult neurogenesis in the limbic system [49]. Accordingly, future work will be needed to evaluate whether chronic SDS exposure (≥ 10 episodes) alters similar molecular markers associated with the birth and survival of neurons in brain regions such as the hippocampus and amygdala, as observed in other rodents exposed to various types of stress [5, 50]. Indeed, expanding our understanding in this area may provide deeper insights into the neurobiological systems involved in stress-induced anxiety-like behavior and their potential translational implications.

A limitation of the present work is that we did not include females in our experimental design, thus limiting the interpretability of our findings to males. However, it is plausible that 10 episodes of SDS could also result in an anxiogenic-like phenotype in adult female prairie voles, as protocols adopting acute (up to 3) SDS episodes have shown to decrease their exploration in the EPM [29]. Also, in future validation studies, the administration of medications with anxiolytic properties (i.e. benzodiazepines, ketamine, or selective serotonin reuptake inhibitors) should be evaluated to determine if they rescue the SDS-induced maladaptive anxiogenic response observed in both adult male prairie voles (Figs. 2, 3) and adult female mandarin voles [41]. This evaluation is necessary to directly provide predictive/pharmacological validity to the vole SDS model for the study of anxiogenic-like phenotypes across a menu of open-exploration behavioral experiments [35, 51]. This is critical because in other rodent species both chronic administration of selective serotonin reuptake inhibitors [31, 52] as well as acute exposure to chlordiazepoxide [14] or ketamine [30, 53] reverse the anxiogenic/social dysfunction reported after social stress.

## CONCLUSIONS

Here, we found that implementing 10 days of SDS, in adult male prairie voles, results in reductions of exploratory behavior in the LDB and EPM tests; thus, capturing an anxiogenic-related outcome [39]. Along with prior work indicating that SDS induces anhedonia-like responses (per decreases in social behavior and sucrose preference), reduces body weight-gain, and impairs spatial memory [22], we provide an experimental stress model that may provide a platform to study the neurobiological mechanisms that underlie social stress-induced maladaptive anxiety-related phenotypes [41]. Lastly, and more importantly, this preclinical vole SDS model has the potential to provide a more translationally relevant approach for preclinical researchers to uncover the behavioral and molecular underpinnings of social stress-induced mood-related illnesses with overlapping anxiety syndromes.

## STUDY FUNDING AND APC FUNDING

This work was funded by the National Institute of General Medical Sciences (SC3GM130467, T34GM145529, and R16GM145552).

## Supplementary Material

OXFNSC-2024-006_Peer_Review_History_kvae012

## Data Availability

Data will be made available upon reasonable request.
