# Supplementary material for: Social defeat stress induces an anxiety-like outcome in male prairie voles (Microtus ochrogaster)
Source: Oxf Open Neurosci. 2024 Nov 23;3:kvae012. doi: 10.1093/oons/kvae012 (PMC11629977; doi:10.1093/oons/kvae012)
Supplement: OXFNSC-2024-006_Peer_Review_History_kvae012 [file oxfnsc-2024-006_peer_review_history_kvae012.zip › OXFNSC-2024-006_Peer_Review_History_kvae012.odt]

OXFNSC-2024-006.R1 Review History

Reviews
Reviewer 1 – Minor Revision
This manuscript investigates the impact of chronic social defeat stress (SDS) on anxiety-like behaviors in male adult prairie voles. The study aimed to validate the SDS paradigm as a preclinical model for studying social stress-induced anxiety. To test this, the authors exposed sexually naive male prairie voles to 10 consecutive days of SDS episodes, involving both physical confrontation and a threat session. Control voles were housed in similar conditions but without experiencing any social conflict. Twenty-four hours after the last SDS episode, the voles were tested for anxiety-like behavior using two established methods: the light/dark box (LDB) test and the elevated plus maze (EPM). The results showed that SDS-exposed voles exhibited significantly increased latency when entering the light compartment of the LDB compared to the control group. They also displayed fewer entries into the open arms of the EPM and spent more time in the closed arms, indicating heightened anxiety. Notably, the observed anxiety-like behavior was not attributed to changes in locomotor activity, as no differences were found between the groups in terms of distance traveled in either test. However, SDS-exposed voles did show a decrease in body weight, a common outcome of stress exposure in rodents. The manuscript is well-written, and the experimental design is sound with appropriate controls. The addition of the anxiety-like behaviors after SDS complements the work that the authors have recently published. Overall, the emphasis on longer stress episodes is important, particularly, as the authors note, parallels and extends the work with mice. I appreciate that the authors already point to future examination of sex differences and pharmacological validation studies in the discussion section.

The following are minor suggestions.

- In the introduction, please provide more context for the choice of 10 SDS episodes. While this was discussed in the discussion section, it would also be relevant to include the rationale in the introduction.

-The authors need to clarify how time spent in the center area of the EPM was scored, or if it was not analyzed.

-The citation "Rodriguez et al, 2024" on page 4 line 73 is missing a period after “al.” It should be "Rodriguez et al., 2024."

Reviewer 2 – Minor Revision
This manuscript examined the effects of chronic social defeat stress (CSDS) on behavioral measures of anxiety-like behaviors (light dark box, elevated plus maze) in adult male prairie voles. The results suggest that CSDS increases anxiety-like behavior in these tests. The study is straight-forward and the manuscript is well-written. I do, however, have some comments and questions that are included below.

General Comments:

1. It would be interesting to compare the effects of CSDS on anxiety-like behaviors in male and female voles. What is the rationale for not including females?
2. Figure 1 suggests that there are vole pups present during the SDS sessions. Please clarify why this is if the experimental rat is an adult male.
3. More details are needed in the Statistical Analysis section. Please indicate how sample sizes were selected and if a power analysis was used. Were normality tests used to verify the distribution of the data and select an appropriate statistical test? Were outliers excluded from analysis? If so, what were the criteria?
4. In mice, CSDS exposure results in a divergent phenotype (some mice are susceptible, some mice are resilient) that is characterized by their performance in a social behavior test (i.e. mice that display social avoidance are considered to be susceptible). Is there evidence for a divergent phenotype following CSDS exposure in voles? Did the authors check for this? This seems like an important factor to consider.
5. It would be useful if the authors could include actual p-values for non-significant effects instead of p > 0.05.
6. The authors identified an interesting future direction to validate CSDS-induced anxiety-like behavior by proposing to test anxiolytic effects of a variety of drugs. It is unclear why this was not incorporated into the current manuscript as it would strengthen both the claims and the novelty of the paper.
7. It would be beneficial for the authors to add in the Discussion what brain areas/systems they think might be associated with the anxiogenic phenotype induced by CSDS.

Decision Letter
Manuscript ID OXFNSC-2024-006 entitled "Social defeat stress induces an anxiety-like outcome in male prairie voles (Microtus ochrogaster)" which you submitted to the Oxford Open Neuroscience, has been reviewed.  The comments of the reviewer(s) are included at the bottom of this letter.

The reviewer(s) have recommended publication, but also suggest some minor revisions to your manuscript.  Therefore, I invite you to respond to the reviewer(s)' comments and revise your manuscript within the next 30 days of todays date.

Please note that this journal operates with transparent peer review. This means that if your submission is accepted for publication, the full peer review history of your article will publish online alongside your article. This includes reviewer comments, editor decision letters, and your author responses.

To revise your manuscript, log into https://mc.manuscriptcentral.com/oxfnsc and enter your Author Centre, where you will find your manuscript title listed under "Manuscripts with Decisions."  Under "Actions," click on "Create a Revision."  Your manuscript number has been appended to denote a revision.

You may also click the below link to start the revision process (or continue the process if you have already started your revision) for your manuscript. If you use the below link you will not be required to login to ScholarOne Manuscripts.

*** PLEASE NOTE: This is a two-step process. After clicking on the link, you will be directed to a webpage to confirm. ***

https://mc.manuscriptcentral.com/oxfnsc?URL_MASK=3015c70b76f34c18919df1e67410b28b


You will be unable to make your revisions on the originally submitted version of the manuscript.  Instead, revise your manuscript using a word processing program and save it on your computer.  Please also highlight the changes to your manuscript within the document by using the track changes mode in MS Word or by using bold or colored text.

Once the revised manuscript is prepared, you can upload it and submit it through your Author Centre.

When submitting your revised manuscript, you will be able to respond to the comments made by the reviewer(s) in the space provided.  You can use this space to document any changes you make to the original manuscript.  In order to expedite the processing of the revised manuscript, please be as specific as possible in your response to the reviewer(s).

IMPORTANT:  Your original files are available to you when you upload your revised manuscript.  Please delete any redundant files before completing the submission.

Because we are trying to facilitate timely publication of manuscripts submitted to the Oxford Open Neuroscience, your revised manuscript should be uploaded within the next 30 days from todays date.  If it is not possible for you to submit your revision in a reasonable amount of time, we may have to consider your paper as a new submission.

Once again, thank you for submitting your manuscript to the Oxford Open Neuroscience and I look forward to receiving your revision.

Response to Reviewers
We have addressed each of the reviewers points individually. Note that corresponding changes to the manuscript are shown in blue.

Reviewer #1:
1.1 “This manuscript investigates the impact of chronic social defeat stress (SDS) on anxiety-like behaviors in male adult prairie voles…The manuscript is well-written, and the experimental design is sound with appropriate controls. The addition of the anxiety-like behaviors after SDS complements the work that the authors have recently published. Overall, the emphasis on longer stress episodes is important, particularly, as the authors note, parallels and extends the work with mice. I appreciate that the authors already point to future examination of sex differences and pharmacological validation studies in the discussion section. The following are minor suggestions. In the introduction, please provide more context for the choice of 10 SDS episodes. While this was discussed in the discussion section, it would also be relevant to include the rationale in the introduction.”

We thank the reviewer for acknowledging that our manuscript is well-written and that our experimental design is valuable to translational research. As recommended, we have now indicated in the Introduction the rationale for using a chronic SDS approach. Specifically, to expand on recent findings indicating that a 10-day SDS protocol results in a depression-relevant phenotype in male prairie voles, as evidenced by decreases of social behavior and sucrose preference, as well as spatial memory impairment [J Affect Disord, 351:833-842, 2024]. However, whether such chronic SDS protocol induces an anxiogenic-like effect in voles was not previously known. Given that depression and anxiety-related phenotypes are highly comorbid, we evaluated whether decreases in exploratory behavior (per the elevated plus-maze and light/dark box tests) would be evident after 10 episodes of SDS in male prairie voles, as commonly reported in mice (see top Paragraph on Page 3).

1.2 “The authors need to clarify how time spent in the center area of the EPM was scored, or if it was not analyzed).”

We have updated the Methods section to clearly indicate that the center area was not analyzed, as it was not classified as part of either the open or closed arms of the EPM (see Page 7, Lines 13-14).

1.3 “The citation "Rodriguez et al, 2024" on page 4 line 73 is missing a period after “al.)”

Thank you for catching the typo. We have updated the references across the manuscript in the new revised version of the paper.

Reviewer #2
2.1 “This manuscript examined the effects of chronic social defeat stress (CSDS) on behavioral measures of anxiety-like behaviors (light dark box, elevated plus maze) in adult male prairie voles. The results suggest that CSDS increases anxiety-like behavior in these tests. The study is straight-forward and the manuscript is well-written. I do, however, have some comments and questions that are included below. It would be interesting to compare the effects of CSDS on anxiety-like behaviors in male and female voles. What is the rationale for not including females?.”

We thank the reviewer for taking the time to evaluate our work and for highlighting that our manuscript is well-written and straightforward. As the reviewer is likely aware, across the SDS literature, experiments are predominantly (> 90%) conducted in male subjects (for review see: Neurosci Biobehav Rev, 145:105032, 2023) wherein defeated males display behaviors associated with mood-related illnesses such as depression and anxiety [Science, 311(5762):864-8, 2006]. Because we follow a hypothesis-driven approach, it is necessary to conduct this study in males first (for direct translational comparisons with what is mostly reported across the literature in mice/rats) including recent published work on the effects of SDS on depression-related behavior in male prairie voles [J Affect Disord, 351:833-842, 2024]. However, we understand that not including females is a limitation, as rightfully noted by the reviewer, and thus address this issue in the Discussion section of the paper (see last paragraph on Page 11). On this note, our laboratory will not work with voles in the future – precluding us from evaluating affect-related effects of SDS in female prairie voles. Specifically (1) because we do not have the funds to conduct such studies, nor (2) access to additional animals from the vole colony at our institution (since other externally funded labs have priority). Nevertheless, we believe that the present work in male voles provides a hypothesis driven approach for other laboratories to conduct such important studies that incorporate females and further expand on the biological factors that mediate SDS-induced anxiety phenotypes (see last two paragraphs on Page 11 of Discussion).

2.2 “Figure 1 suggests that there are vole pups present during the SDS sessions. Please clarify why this is if the experimental rat is an adult male.".

As previously published, we used adult, actively breeding, paired-bonded male voles as aggressors (with potential pups in their home cage); specifically, to ensure defensive and consistent aggression towards the experimental intruder adult vole [J Affect Disord, 351:833-842, 2024]. To avoid confusion, we now clearly describe this approach in the “Aggression Screening in Sexually Experienced Male Voles” section of the paper (Page 4, Lines 6-9; see also Social Defeat Stress section on Page 5, and Figure Caption 1). Although we did not keep actual records of the number of pair-bonded couples with offspring present, most pair bonded couples indeed have pups in their home-cage (~70%). As a result of this, we obtained consistent attack latencies across each aggressor (within 30 seconds), thus controlling for offensive quality and reliability, as previously described and quantified [Psychoneuroendocrinology, 113, 104542, 2020].

2.3 “More details are needed in the Statistical Analysis section. (a) Please indicate how sample sizes were selected and if a power analysis was used. (b) Were normality tests used to verify the distribution of the data and select an appropriate statistical test? (c) Were outliers excluded from analysis? If so, what were the criteria?.”

2.3a Prairie voles are not commercially available, and accordingly, the number of animals per group was designated based on the limited availability of voles from the breeding colony at our home institution. Independent of this, and for transparency reasons, we state the number of voles per experimental condition in both the Results section of the paper (Pages 8-9) and in Table 1.
2.3b No tests of normality were conducted prior to the analyses, given the restricted number of animals that were available to us. Nevertheless, individual data points are available within each experimental group across the Figures (Figs. 2-4). Also, we report specific t and p values, degrees of freedom, individual n’s per experimental condition, and effect sizes (partial eta squared) across the manuscript (see Results section on Page 8-9, and/or Table 1).
2.3c No data points were excluded from any analyses, particularly because no outliers were detected according to Grubbs’ tests. However, a total of four animals from the SDS groups died prior to behavioral assessment (two from each cohort). This outcome aligns with our experience and findings reported in the literature, which indicate that approximately 20% of animal attrition is typical/expected in SDS experiments. We now note (1) animal attrition and (2) that outlier detection analyses were conducted in the Statistical Analysis section of the paper (see end of Page 7 and top of Page 8).

2.4 “In mice, CSDS exposure results in a divergent phenotype (some mice are susceptible, some mice are resilient) that is characterized by their performance in a social behavior test (i.e. mice that display social avoidance are considered to be susceptible). Is there evidence for a divergent phenotype following CSDS exposure in voles? Did the authors check for this? This seems like an important factor to consider.”

The reviewer makes a great observation. It is well established that SDS results in a subpopulation of animals classified as resilient (versus susceptible) in both mice [Cell, 131(2):391-404, 2007] and rats [Endocrinology, 151(4):1795-1805, 2010]. Thus, it is possible that SDS results in a subpopulation of resilient animals in prairie voles as well. However, given that such studies (evaluating resilience versus susceptibility) require a very large number of animals per experimental condition [Cell, 131(2):391-404, 2007] we did not classify animals as resilient or susceptible post-SDS in the present manuscript. Also, because the established resiliency screening method is validated via the social interaction test [Cell, 131(2):391-404, 2007] and in the present work we did not evaluate sociability. Lastly, and more importantly, the literature indicates that rodents classified as resilient exhibit an anxiogenic-like phenotype [Cell, 131(2):391-404, 2007; Biol Psychiatry, 88(5):381-391, 2020] per traditional behavioral methods including the elevated plus maze [Neurosci Bull, 38(1):16-28, 2022]. Because we are only evaluating anxiety-related outcomes (elevated plus maze and light/dark box) we do not believe we could provide a robust argument to evaluate and determine whether SDS induces a divergent phenotype in the present work.

2.5 “It would be useful if the authors could include actual p-values for non-significant effects instead of p > 0.05.”

We have complied with the reviewer’s request. We now provide exact p values for non-significant effects in the manuscript (see Results section on Pages 8-9 and Table 1).

2.6 “The authors identified an interesting future direction to validate CSDS-induced anxiety-like behavior by proposing to test anxiolytic effects of a variety of drugs. It is unclear why this was not incorporated into the current manuscript as it would strengthen both the claims and the novelty of the paper.”

We agree with the reviewer – that adding additional pharmacological experiments to the present investigation would strengthen the impact of the paper. Unfortunately, our laboratory will not be working with voles in the future, particularly, because we do not have the funds to conduct such studies. Furthermore, we do not have access to additional animals from the vole colony at our institution, as priority is given to other externally funded laboratories. For these reasons we provide future directions that would directly expand and validate how SDS in prairie voles induces an anxiogenic phenotype – specifically, studies that will address pharmacological validity as well as the potential biological factors that mediate SDS-induced anxiety-like phenotypes (see second and third paragraph on Page 11).

2.7 “It would be beneficial for the authors to add in the Discussion what brain areas/systems they think might be associated with the anxiogenic phenotype induced by CSDS.”

As recommended, we now briefly address this issue in the Discussion section of the paper (see second paragraph on Page 11, Lines 3-14). Specifically, we highlight how serum corticosterone and areas of the limbic system (hippocampus and amygdala) likely are dysregulated by SDS exposure in prairie voles, thus, underlying the anxiogenic phenotype observed, as in other rodents [ILAR J, 55(2):221-32, 2014; Biol Psychiatry, 88(5):381-391, 2020; Int J Mol Sci, 24(13):11193, 2023].

Decision Letter
It is a pleasure to accept your revised manuscript entitled "Social defeat stress induces an anxiety-like outcome in male prairie voles (Microtus ochrogaster)" in its current form for publication in the Oxford Open Neuroscience. The comments of the reviewer(s) who reviewed your manuscript are included at the foot of this letter.

Please note that this journal operates with transparent peer review. This means that  the full peer review history of your article will publish online alongside your article. This includes reviewer comments, editor decision letters, and your author responses.

Next steps
You will receive an email from no-reply@scipris.com within roughly one week. This is your invitation to sign up for an account with SciPris, Oxford University Press’ author portal hosted by Aptara. You will need to create an account if you do not already hold one. Please register or log into your account and follow the online instructions which will guide you through signing your licence and paying the APC. The email and the portal have clearly signposted support options if you need any help during this process.

Please note that SciPris is a completely different system from ScholarOne, so your credentials to submit your manuscript here will not work there. Once you’ve created a SciPris account, you will be able to use it whenever you publish with Oxford Open Neuroscience or any OUP journal. Please note that OUP will only ever request payment for applicable fees be made via SciPris or to an OUP bank account. If you ever have concerns about the legitimacy of a request, please do not hesitate to contact a customer services agent via the SciPris portal or directly via oupsupport@scipris.com.

Thank you for your fine contribution.  On behalf of the Editors of the Oxford Open Neuroscience, we look forward to your continued contributions to the Journal.


Reviewer: 1

Comments to the Author
The authors have addressed my concerns. I have no further comments.

Reviewer: 2

Comments to the Author
I am satisfied with the authors' response to my comments.
